# Bacteriophage Combinations Significantly Reduce Clostridium difficile Growth *In Vitro* and Proliferation *In Vivo*

**DOI:** 10.1128/AAC.01774-15

**Published:** 2016-01-29

**Authors:** Janet Y. Nale, Janice Spencer, Katherine R. Hargreaves, Anthony M. Buckley, Przemysław Trzepiński, Gillian R. Douce, Martha R. J. Clokie

**Affiliations:** a Department of Infection, Immunity and Inflammation, University of Leicester, Leicester, England, United Kingdom; b Institute of Infection, Immunity and Inflammation, College of Medical, Veterinary and Life Sciences, University of Glasgow, Glasgow, Scotland, United Kingdom

## Abstract

The microbiome dysbiosis caused by antibiotic treatment has been associated with both susceptibility to and relapse of Clostridium difficile infection (CDI). Bacteriophage (phage) therapy offers target specificity and dose amplification *in situ*, but few studies have focused on its use in CDI treatment. This mainly reflects the lack of strictly virulent phages that target this pathogen. While it is widely accepted that temperate phages are unsuitable for therapeutic purposes due to their transduction potential, analysis of seven C. difficile phages confirmed that this impact could be curtailed by the application of multiple phage types. Here, host range analysis of six myoviruses and one siphovirus was conducted on 80 strains representing 21 major epidemic and clinically severe ribotypes. The phages had complementary coverage, lysing 18 and 62 of the ribotypes and strains tested, respectively. Single-phage treatments of ribotype 076, 014/020, and 027 strains showed an initial reduction in the bacterial load followed by the emergence of phage-resistant colonies. However, these colonies remained susceptible to infection with an unrelated phage. In contrast, specific phage combinations caused the complete lysis of C. difficile *in vitro* and prevented the appearance of resistant/lysogenic clones. Using a hamster model, the oral delivery of optimized phage combinations resulted in reduced C. difficile colonization at 36 h postinfection. Interestingly, free phages were recovered from the bowel at this time. In a challenge model of the disease, phage treatment delayed the onset of symptoms by 33 h compared to the time of onset of symptoms in untreated animals. These data demonstrate the therapeutic potential of phage combinations to treat CDI.

## INTRODUCTION

Clostridium difficile (Peptoclostridium difficile) is responsible for approximately 39% of the cases of antibiotic-associated diarrhea in the Western world, causing death in 10% of patients (1). Two of the main problems associated with disease prevention include the diverse virulent ribotypes (strains) that cause infections that are resistant to and respond differently to antibiotic treatment and the production of spores that are impervious to chemical decontamination (2–4). The prevalence of C. difficile, the cost of infection control, and the challenge of finding alternative treatments all contribute to the fact that infection with this organism remains a significant clinical and financial burden to the health care system (5, 6).

Acute C. difficile infection (CDI) is treated using antibiotics, with three options currently being available: metronidazole, vancomycin, and fidaxomicin (7–9). Metronidazole and vancomycin are the most commonly prescribed and are given either individually or in combination. This strategy has been implemented despite the fact that metronidazole is not effective against all ribotypes and that vancomycin is the antibiotic of last resort against many other multidrug-resistant pathogens and, thus, its prolonged use is discouraged (8, 9). Fidaxomicin has an effect similar to that of vancomycin, but the high cost associated with this antibiotic also limits its clinical use (10, 11). Importantly, a major disadvantage associated with antibiotic use for the treatment of CDI is the risk of exacerbating further the dysbiosis (imbalance) of the microbiota. This results in normal gut commensals being reduced or removed, thus allowing C. difficile to effectively colonize this niche (12). Furthermore, antibiotic therapy of this infection is often associated with high recurrence rates and provides an opportunity for antibiotic resistance to emerge (13). This negative association with antibiotic use further highlights the need to identify alternative, more effective strategies to treat CDIs.

Phage therapy offers several advantages over therapy with conventional antibiotics (12, 14, 15). Phages are viruses which specifically infect bacteria, and phage therapy exploits the specific nature of these phage-bacterium interactions (16). The potential use of phages for the treatment of infectious diseases has been extensively discussed, and phages have been shown to effectively reduce or remove specific bacteria responsible for a wide range of infections in both animals and humans (14, 17–19). Pertinent to CDI is the minimal deleterious effect that they have on the gut microbiota; consequently, their use prevents further dysbiosis. Furthermore, phages replicate in a self-limiting manner at a localized site of infection; thus, their effective dosage is amplified (20). A third useful attribute is their ability to penetrate the complex biofilm environments found in C. difficile-associated pseudomembranous plaques (19, 21, 22).

Phage therapy is used widely in Russia and Georgia, where phage cocktails targeting many bacterial species, including Staphylococcus, Streptococcus, Pseudomonas, Proteus, and Escherichia coli, are available as over-the-counter pharmaceutical products (16, 23). In Poland, phage therapy has been successfully used to treat chronic and antibiotic-resistant suppurative infections (24). Furthermore, there is an increasing interest in their potential use in many Western countries, and several clinical trials are in preparation (23, 25, 26). Interestingly, phages have been approved for use in food products, demonstrating their increasing acceptability as a safe and useful antimicrobial agent (27, 28).

To date, treatments based on phage therapy have not been developed to treat CDIs. This is the case despite recent recommendations that this type of therapy be offered as an alternative to conventional antibiotic treatment for CDI (12, 14, 15). The reason for this lack of development is largely because C. difficile has not been a target pathogen in the countries where phage therapy is used. Advances in this area have also been limited by the lack of strictly virulent phages that infect this pathogen and by the fact that the isolation of phages for this organism can be challenging, so in some studies, only 1 or 2 C. difficile phages have been isolated and tested (29–31). In species where no lytic phages have been identified, the only pragmatic option for the development of phage therapeutics is to test the efficacy of multiple wild-type phages to determine their efficacy *per se* and, potentially, to develop the lysogenic phages further using engineering approaches.

C. difficile phages that can access the lytic life cycle have been isolated both in our laboratory and elsewhere (32–37), but to date, no studies have described the use of phage cocktails/combinations against C. difficile (14). Here, we determined the activity of seven phages individually and in combination against clinically relevant ribotypes of C. difficile. Using individual phages and phage combinations, we demonstrate the synergy of phage combinations for the effective clearance of C. difficile. Optimized phage combinations were also shown to reduce colonization and disease symptoms and extend longevity in a hamster model of CDI. Our results support the potential application of phage combinations for the targeted eradication of CDI.

## MATERIALS AND METHODS

### Bacterial strains and culture conditions.

The C. difficile isolates used in this study include those from our laboratory collection and those kindly provided by Richard Stabler (London School of Hygiene and Tropical Medicine, London, United Kingdom), Trevor Lawley (Wellcome Trust Sanger Institute, Cambridge, United Kingdom), and Mark Wilcox (University of Leeds, Leeds, United Kingdom), who also donated the U.S. strains. The Australian strains were a kind gift from Dena Lyras (Monash University, Australia) and Tom Borody (Center for Digestive Diseases, Australia). C. difficile strains were subcultured on brain heart infusion (BHI) agar (Oxoid, Ltd., United Kingdom) supplemented with 7% defibrinated horse blood (TCS Biosciences, Ltd., United Kingdom). The strains were preserved in Protect bacterial preservers (Abtek Biologicals Ltd., Liverpool, United Kingdom) and stored at −80°C. Strains were characterized using the primers 5′-TTGAGCGATTTACTTCGGTAAAGA-3′ and 5′-CCATCCTGTACTGGCTCACCT-3′ targeting the C. difficile 16S rRNA gene (38) and capillary gel electrophoresis-based PCR ribotyping with primers 5′-GTGCGGCTGGATCACCTCCT-3′ and 5′-CCCTGCACCCTTAATAACTTGACC-3′ to amplify the 16S-23S rRNA genes (39). Data analysis was performed using Peak Scanner software (Applied Biosystems, United Kingdom) and the Multivariate statistical package (version 3.1).

### Phage isolation and propagation and host range analysis.

Phages were isolated from the enrichment of estuarine samples and the induction of estuarine isolates (40, 41). The phages were initially screened for activity, performed by the observation of lysis on a lawn of bacteria (42). Briefly, 3 ml of a 0.5% BHI agar overlay containing 0.01 M CaCl_2_, 0.4 M MgCl_2_, and 250 μl of an overnight culture of indicator bacteria grown in fastidious anaerobic broth (FAB; BioConnections, Leeds, United Kingdom) was prepared. Aliquots of 10 μl of the phage samples were applied on the agar overlay and allowed to dry for ∼2 min at room temperature before incubation at 37°C anaerobically overnight. Phages were made clonal through subjection to five rounds of purification using a plaque assay as previously described (34, 41). The morphology of the phages was identified using transmission electron microcopy (TEM) analysis (43). The presence of temperate phages within the manufacturing hosts was ascertained by subjecting the cultures to 3 μg/ml norfloxacin (Sigma-Aldrich, Dorset, United Kingdom) or mitomycin C (Fisher Scientific, Loughborough, United Kingdom) and monitoring for spontaneous release using TEM. The host range of the seven phages was tested as described above using 10^8^ PFU/ml of lysates (42).

### Phage DNA extraction, sequencing, and phylogenetic analysis.

Genomic DNA was extracted from the crude lysates using a modified phenol-chloroform-isoamyl alcohol method. Briefly, the 1-ml lysates with 10^9^ PFU/ml of phages were treated with 12.5 mM MgCl_2_ (Acros Organics, NJ, USA), 0.8 U/ml of DNase (Sigma, USA), and 0.1 mg/ml of RNase (Sigma, USA) (final concentrations) and incubated at room temperature for 1 h to eliminate bacterial DNA. Afterwards, EDTA (Sigma, USA), proteinase K (Fisher Scientific, Germany), and sodium dodecyl sulfate (Fisher Scientific, United Kingdom) were added to final concentrations of 20 mM, 0.5 mg/ml, and 0.5%, respectively, before incubation at 55°C for an additional hour to digest the phage capsid. DNA material was extracted with an equal volume of phenol-chloroform-isoamyl alcohol (25:24:1, vol/vol/vol) and centrifuged at 15,000 × *g* for 5 min. The aqueous layer obtained was treated with 0.3 M sodium acetate (Fisher Scientific, United Kingdom) and 2 volumes of ice-cold ethanol to precipitate the DNA. After incubation for 10 min on ice, the DNA was eluted by centrifugation at 21,000 × *g* for 20 min, and the resultant pellet was washed once with 0.5 ml of 70% ethanol before it was dissolved in 5 mM Tris HCl. The DNA quality and quantity were analyzed using a NanoDrop 2000 spectrophotometer and a Qubit double-stranded HS assay kit on a Qubit fluorometer (Thermo Scientific, United Kingdom), respectively, before the DNA was sequenced using Illumina HiSeq sequencing. Gene prediction was conducted using the Rapid Annotation Using Subsystem Technology (RAST) server (44) and visualized with Geneious R8 software (45). Minor capsid genes from all the sequenced genomes and C. difficile phage sequences were extracted from the National Center for Biotechnology Information (NCBI) database. The evolutionary history of the minor capsid genes was inferred using the maximum likelihood method in MEGA (version 6.06) software, and the tree was visualized with the FigTree (version 1.4.2) program.

### Phage-bacterium coinfection assay.

High plaquing efficiencies by five of the seven phages (phiCDHM1 to phiCDHM3, phiCDHM5, and phiCDHM6) were observed on strains CD105HE1 and CD105LC2. Similarly, phiCDHS1 propagated efficiently on strain R20291. These phages and C. difficile strains were selected for further *in vitro* analysis. To prepare liquid cultures, a single colony from BHI agar-supplemented medium was inoculated into FAB and incubated overnight. Fresh cultures were prepared by subculturing 1% of the overnight culture into prereduced BHI broth; growth was monitored by measuring the optical density (OD) at 550 nm (OD_550_). Once the cultures reached an OD of 0.2 ± 0.01 (10^7^ CFU/ml cells), phage lysates were added at a multiplicity of infection (MOI) of 10. The MOI remained constant even when combinations of phages were prepared by combining equal amounts of phages. Cultures were monitored over 24 h, and viability counts were recorded hourly until 5 h and then again at 24 h.

### Analysis of phage-resistant and lysogenic bacteria.

To isolate phage-resistant and lysogenic bacterial colonies, a 10-fold dilution of the bacterial culture at an OD_550_ of 0.2 ± 0.01 was prepared in BHI broth. Approximately 10 μl of the diluted bacterial culture was applied on 3-ml BHI semisolid agar overlays on brucella agar (Oxoid, United Kingdom) supplemented with 5% blood, 5 mg/liter hemin, and 1 mg/liter vitamin K (46). Similarly, the diluted cultures were also applied to semisolid agar overlays containing 250 μl of 10^9^ PFU/ml of the most active phages (phiCDHM1, phiCDHM2, or phiCDHM3). Colonies recovered from the phage-treated media were subsequently purified from phage contamination by serial passage on BHI agar plates supplemented with 7% defibrinated horse blood on at least three occasions. All resistant colonies from the phage treatments were identified to be C. difficile using 16S rRNA analysis (38). The bacteria were further characterized for the presence or absence of the phages using primers specific for the NACHT gene of phages phiCDHM1 and phiCDHM3 (5′-GAAGCACTTGGAAAACAAAGG-3′ and 5′-CGCAAGAAGCATCAAAAACA-3′) (14) and the tail protein gene of phage phiCDHM2 (5′-GAGGGCAGGAATAAGAAAAGC-3′ and 5′-GATTCCCTATCCTCAACTACGC-3′). The sensitivity of resistant, lysogenic, and wild-type bacteria to phages phiCDHM1 to phiCDHM3, phiCDHM5, and phiCDHM6 was determined using a spot test with serially diluted phage lysates.

### Efficacy of phage treatment on colonization *in vivo*.

**FIG 1:**
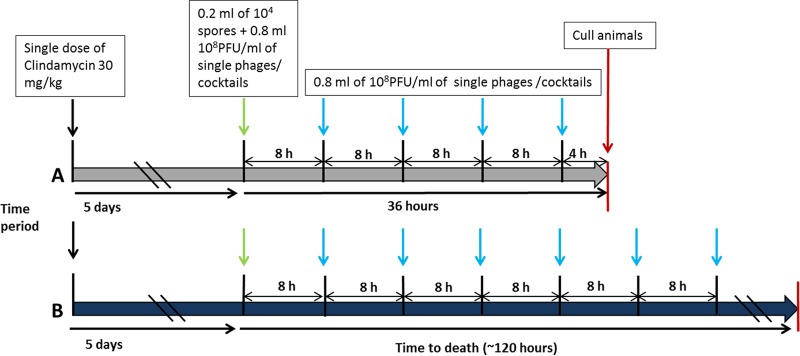
Schematic representation of timing of infection and phage dosing *in vivo*. (A) To determine the impact of the phages on colonization, animals were infected and dosed with a single phage or a combination of phages every 8 h for 36 h. Animals culled at this point showed no symptoms, even though they subsequently showing high levels of bacterial colonization. No toxin was detected in any animal at this time point. (B) To determine the protective efficacy of the phages, animals were infected and treated with the phages as described in the legend to panel A. Treatment was continued until the body temperature of the animal reached 35°C, at which point the animal was culled and the level of colonization of tissue was determined. All animals were made sensitive to C. difficile by oral treatment with clindamycin 5 days prior to challenge with bacterial spores.

The efficacy of phage treatment was assessed *in vivo* using the Syrian Golden hamster model of acute C. difficile infection. This model reflects many of the clinical features of the disease, including toxin-mediated diarrhea and tissue pathology (29, 30, 47). All procedures were conducted in strict accordance with the Animals Act (Scientific Procedures, 1986) and were approved by the Home Office, United Kingdom (project license number PPL60/4218). Female Syrian Golden hamsters that weighed approximately 100 g (bred in-house) were housed individually in sterilized cages and given irradiated water and pelleted standard B&K rat and mouse food (DBM, Scotland) *ad libitum*. Each animal received 30 mg of clindamycin phosphate per kg of body weight orogastrically in a single dose administered 5 days before challenge. At approximately 10 min prior to challenge or subsequent phage treatment, the animals were treated with 400 μl 1 M sodium bicarbonate (Sigma, United Kingdom) to reduce stomach acid. Animals were infected with 0.2 ml of 10^4^ CFU/ml spores of the C. difficile CD105HE1 strain (Fig. 1A and B) and subsequently treated with 0.8 ml of 1 × 10^8^ PFU/ml as either a single phage or a combination of phages. The first dose was given at the time of challenge, with subsequent doses being given every 8 h until the scheduled endpoint of 36 h (Fig. 1A). At this point the animals were culled and the impact of phage treatment was quantified by enumeration of the total bacterial load (spores and vegetative cells) in the cecum and colon. This was achieved by removing and opening each section longitudinally. The contents were removed by gentle washing in 10 ml phosphate-buffered saline (PBS) (lumen-associated [LA] bacteria), and the viable counts of the recovered bacteria were determined by plating serial 10-fold dilutions on selective cefoxitin-cycloserine-egg yolk (CCEY) agar (BioConnections, Leeds, United Kingdom) plates. To evaluate the level of more intimately associated organisms, the tissue was further washed in 10 ml PBS and homogenized in 5 ml of PBS for 1 min using a Lablender 80 stomacher (Seward Medical, United Kingdom) (tissue-associated [TA] bacteria), and the homogenates were again diluted and plated as described above. To determine the numbers of spores present, the same samples were heated for 15 min at 65°C, and the numbers of spores present were determined by serial dilution and plating of these samples. Results are shown as the mean number of bacteria recovered from six to eight animals.

For analysis of disease onset, two groups of six animals each were treated and infected as described above, except that treatment with phages continued every 8 h until the experimental endpoint (Fig. 1B). Animals were monitored throughout using a combination of observation and telemetry to measure the core body temperature. Previous experience has shown that animals are unable to recover from disease when the core body temperature drops by 2°C to 35°C, and this decrease provides the experimental endpoint.

Statistical analyses were performed using GraphPad Prism (version 6) software, and a Mantel-Cox log-rank statistical test was used to determine significant differences in survival times between hamsters infected with the different C. difficile strains. *P* values of ≤0.05 were considered significant.

## RESULTS

### Phage classification and relationships.

**FIG 2:**
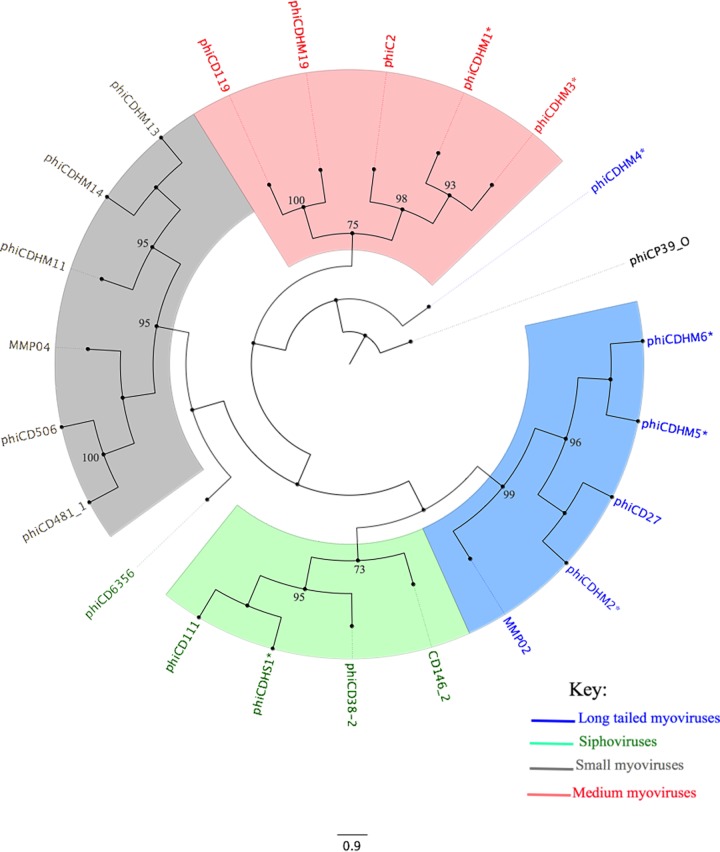
Phylogenetic analysis of the minor capsid gene of C. difficile phages. The evolutionary history of C. difficile phages based on the capsid gene was inferred using the maximum likelihood method based on the Whelan and Goldman frequency model (74). An initial tree(s) for the heuristic search was obtained by applying the neighbor-joining method to a matrix of pairwise distances estimated using a JTT model. The percentage of trees in which the associated taxa clustered together is shown next to the branches. The tree is drawn to scale, with branch lengths being measured as the number of substitutions per site. The analysis involved 22 C. difficile phage minor capsid sequences, including those of the 7 phages examined in this study (*) and one C. perfringens phage, phiCP39_0, as an outgroup. The clades are consistent with the phage morphologies represented by blue (long-tailed myoviruses), green (siphoviruses), gray (small myoviruses), and red (medium myoviruses) (75). The accession numbers of all the sequences used are found in Table S1 in the supplemental material. Evolutionary analyses were conducted in the MEGA (version 6) program (76) and visualized with the FigTree (version 1.4.2) program.

Seven phages (phiCDHM1 to phiCDHM6, and phiCDHS1) were isolated and examined in this study (Table 1). The phages were characterized according to their morphology, host range, and molecular diversity. Six (phiCDHM1 to phiCDHM6) are myoviruses, and one (phiCDHS1) is a siphovirus (Table 1). Phylogenetic analysis of the putative minor capsid gene revealed that the phages are diverse but form discrete groups consistent with their morphology (Fig. 2; see also Table S1 in the supplemental material). The terminal groups to which the phages belong are supported by high bootstrap values (>75%), but the relatedness of the groups varies. Further genomic analysis has shown that, consistent with all other known C. difficile phages, the genomes of these phages encode integrase genes, and thus, the phages can potentially access the lysogenic life cycle. However, all seven phages clearly access the lytic cycle following initial infection of clinically relevant isolates.

**TABLE 1:**
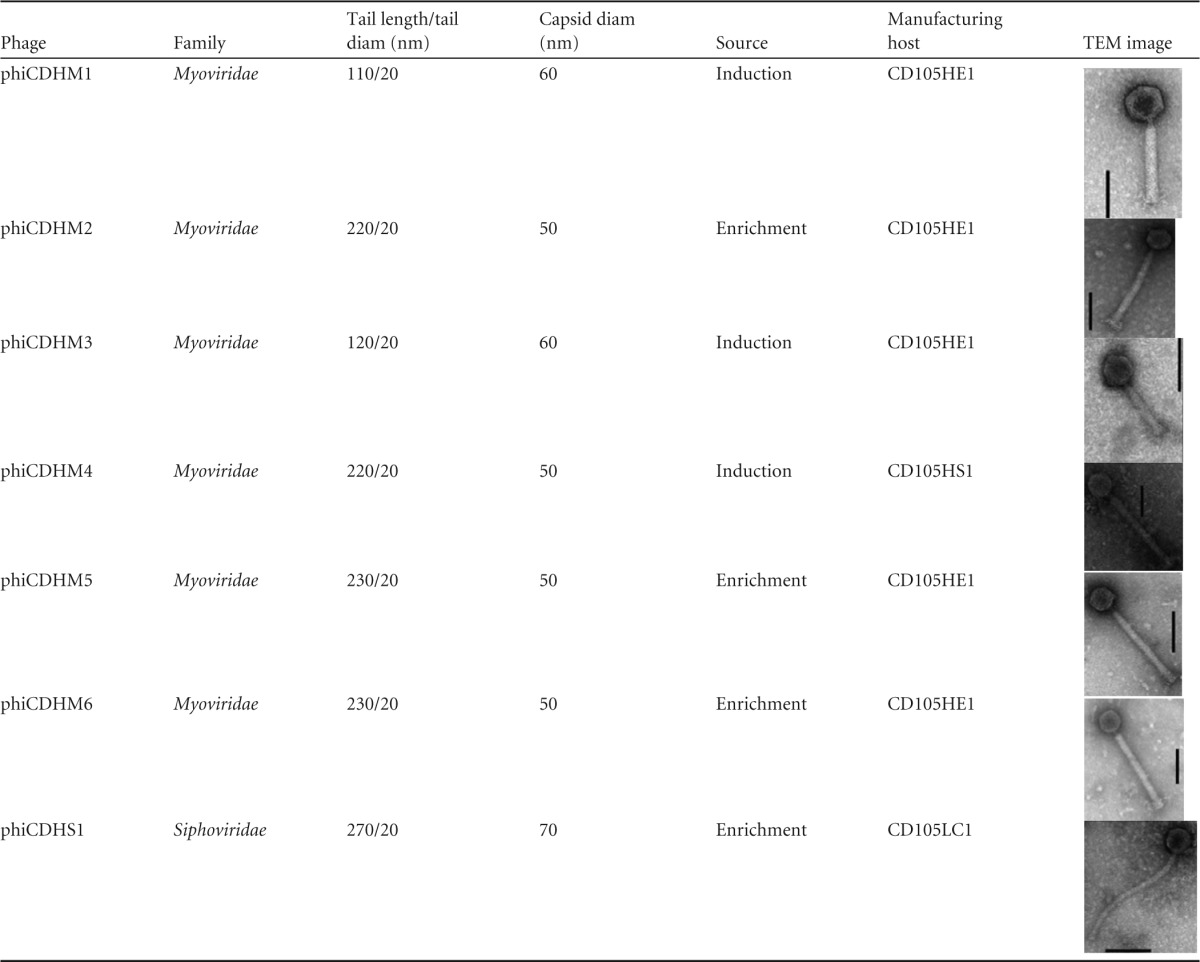
Characteristics of the seven phages examined in this study^*a*^

a The phages were isolated from enrichment of estuarine samples or induction of C. difficile isolates from the same environment, as indicated. Six of the phages (phiCDHM1 to phiCDHM6) are myoviruses, while phiCDHS1 is a siphovirus. Three phages (phiCDHM1, phiCDHM3, and phiCDHM4) were induced from environmental C. difficile isolates, while phiCDHM2, phiCDHM5, phiCDHM6, and phiCDHS1 were isolated from enrichment of the samples. Bars, 60 nm.

### Phage propagation.

To produce an optimized product for therapeutic development, phages were propagated on specific bacterial strains that generate high titers. These bacterial strains are referred to as the manufacturing hosts. Three C. difficile isolates, CD105HE1 (ribotype 076, equine isolate) (48), CD105HS1 (ribotype 012, environmental isolate) (40), and CD105LC1 (ribotype 027, human isolate) (49), were required to propagate the seven phages (Table 1). Five of the phages (phiCDHM1 to phiCDHM3, phiCDHM5, and phiCDHM6) were propagated on CD105HE1 and, when optimized procedures were followed, produced phages at titers of 10^10^ PFU/ml. phiCDHM4 was propagated on CD105HS1, and phiCDHS1 was propagated on CD105LC1. Both produced phages at titers of 10^9^ PFU/ml.

The manufacturing hosts were characterized according to the content and activity of their prophages, to determine if any such prophages contributed to the activity of the final product. Previously, genome sequencing has shown that the genomes of CD105HS1, CD105HE1, and CD105LC1 encode one prophage each (40, 48, 49). However, no prophages were spontaneously released under the optimized conditions used during the propagation of any of the seven phages. In contrast, the prophages could be induced using 3 μg/ml of mitomycin C or norfloxacin (see Table S2 in the supplemental material). Fortunately, none of the prophages from the manufacturing hosts could infect their host strain or any of the clinically relevant bacterial isolates tested.

### Phage host ranges.

For a phage-based product to be effective, it must target the most clinically relevant strains of C. difficile. To determine the host range of the phages, the most prevalent disease-causing ribotypes from the United Kingdom, the United States, and Australia (see Fig. S1 and Table S3 in the supplemental material) were identified through an extensive literature search, with 21 clinically relevant ribotypes being identified on the basis of prevalence and severity in these three countries. They were ribotypes 001, 002, 003, 005, 012, 013, 014/020, 015, 017, 018, 023, 026, 027, 078, 081, 087, 106, 107, 126, 127, and 262 (3, 6, 50–55). In order to assess the host range on a wide range of C. difficile strains, 80 isolates representing the 21 ribotypes were obtained and used as targets in host range assays (see Fig. S1 and Table S3 in the supplemental material).

The ability of the phages to lyse the 80 isolates of C. difficile was tested using 10-μl stocks of 10^8^ PFU/ml inoculated on lawns of bacteria. While the killing spectrum of individual phages varied between ribotypes, the phages successfully lysed 18 (86%) of the ribotypes and 62 (78%) of the C. difficile isolates tested. C. difficile isolates from three ribotypes, 017, 126, and 262, were not sensitive to any of the phages (see Table S3 in the supplemental material). However, these ribotypes currently contribute to less than 6% of cases of clinical disease in the United Kingdom (55) and to even fewer cases in the United States and Australia. Work is ongoing to increase the coverage through the isolation of phages for these ribotypes.

**FIG 3:**
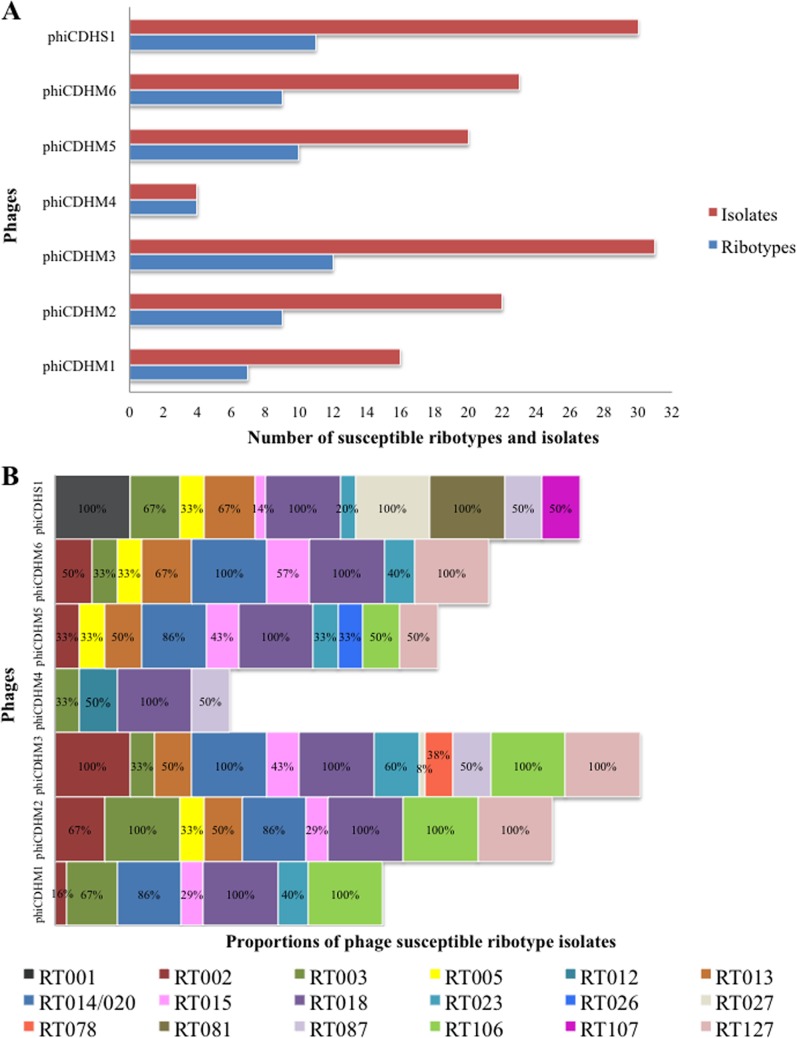
Host range analysis of the seven phages against 80 strains and 21 clinically relevant C. difficile ribotypes found in the United Kingdom, the United States, and Australia. The host range of the phages was determined using a spot test with 10 μl of 10^8^ PFU/ml of phage stocks on agar overlays containing cultures of the hosts. (A) Total number of susceptible ribotypes and strains infected by the phages; (B) percentage of strains per ribotype (RT) susceptible to infection by each of the phages.

The efficacy of phage lysis varied considerably. Three phages, phiCDHM3, phiCDHS1, and phiCDHM5, were able to lyse the most C. difficile isolates and ribotypes: 31 isolates of 12 ribotypes, 30 isolates of 11 ribotypes, and 20 isolates of 10 ribotypes, respectively. Similarly, phiCDHM2, phiCDHM6, and phiCDHM1 had relatively wide host ranges, lysing 22 C. difficile isolates of 9 ribotypes, 23 isolates of 9 ribotypes, and 16 isolates of 7 ribotypes, respectively. Phage phiCDHM4 had the narrowest host range and could lyse just 4 ribotypes, although interestingly, it was the only phage to lyse ribotype 012 and thus provides coverage for this important strain (Fig. 3A and B; see also Table S3 in the supplemental material).

### Effect of phage treatments on C. difficile.

In order for phages to be effective therapeutics, they must significantly reduce the bacterial load *in vivo* by either eliminating all bacteria or reducing them to a level whereby the immune system can subsequently clear the organism. To determine the lytic capacity of individual phages and combinations of phages, we compared the abilities of five phages to lyse bacterial cultures *in vitro* over a 24-h time period. The phages studied were phiCDHM1 to phiCDHM3, phiCDHM5, and phiCDHM6, all of which can be propagated on the same host, CD105HE1. Having a common manufacturing host ensured that any lytic activity could be attributed to the specific phage and not to possible differences conferred by the host bacterial strain. The activities of five of the phages were also determined using the clinical C. difficile isolate CD105LC2 (ribotype 014/020). This strain was selected for analysis as it is highly prevalent in the clinical setting and the strain was shown to be susceptible to infection by the same five phages that infect CD105HE1.

### Effects of single-phage treatments.

**FIG 4:**
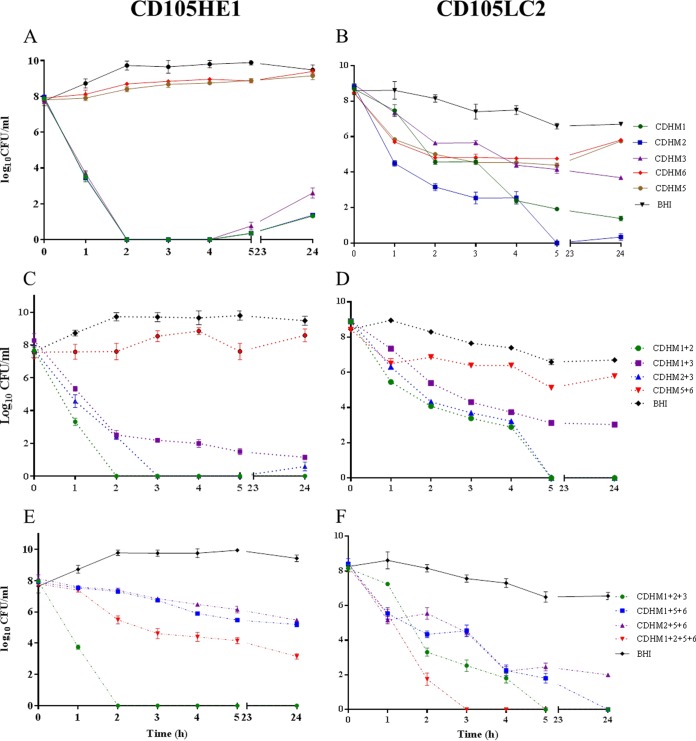
Number of viable C. difficile cells following treatment with phages *in vitro*. Phage infection was carried out at an MOI of 10. (A, C, and E) CD105HE1 treated with a single phage and two- and three- or four-phage combinations, respectively; (B, D, and F) treatment of CD105LC2 with a single phage and two- and three- or four-phage combinations, respectively. Viable C. difficile cells in the culture medium were enumerated at the different time points. Error bars represent the standard errors of the means (SEMs) of three replicates.

Initially, phages were tested in liquid culture, using an MOI of 10, and the efficacy of killing was determined by testing the viable bacterial counts (numbers of CFU per milliliter) in the culture. Most phages initially cleared the bacterial culture, but after 24 h bacterial regrowth occurred (Fig. 4A). phiCDHM1 and phiCDHM2 were more efficient than phiCDHM3, but phiCDHM5 and phiCDHM6 were less efficient than the other three phages at clearing the cultures, with no differences between the treated and control cultures being observed by 24 h.

Replication and lysis by the phages took longer in clinical isolate CD105LC2 (ribotype 014/020) than in the manufacturing host (CD105HE1), with culture clearance not being observed until 5 h postinfection. phiCDHM2 was the most effective phage, reducing bacterial counts to below the limit of detection by 5 h, although regrowth was observed by 24 h (Fig. 4B). Treatment with phiCDHM1 and phiCDHM3 also proved effective, with the viability of the culture being reduced by 6 and 4 log units, respectively, at 5 h. As observed previously, phiCDHM5 and phiCDHM6 proved to be the least effective phages, facilitating only a 2-log-unit reduction in bacterial numbers at 5 h.

### Optimization of two-phage combinations.

To determine the optimal phage combinations, three of the most effective phages and two less effective phages were systematically paired together as follows: phiCDHM1-phiCDHM2, phiCDHM1-phiCDHM3, phiCDHM2-phiCDHM3, and phiCDHM4-phiCDHM5 (Fig. 4C and D).

The most effective lysis was observed using a combination of phages phiCDHM1 and phiCDHM2, which completely lysed CD105HE1 at 2 h postinfection with no regrowth after 24 h. Although bacteria could not be detected at 3 h postinfection with phiCDHM2-phiCDHM3, the organisms were detected 2 h later. In contrast phiCDHM1-phiCDHM3 and phiCDHM5-phiCDHM6 were not as effective (Fig. 4C).

Interestingly, the same pattern of activity was also observed on strain CD105LC2, with phiCDHM1-phiCDHM2 and phiCDHM2-phiCDHM3 causing complete lysis of the bacteria with no subsequent bacterial regrowth (Fig. 4D). In contrast phiCDHM1-phiCDHM3 and phiCDHM5-phiCDHM6 were less efficient and caused 4- and 2-log-unit reductions, respectively.

### Optimization of three- and four-phage combinations.

The results of these paired phage assays informed the *in vitro* testing choice for three three-phage combinations and one four-phage combination. For the three-phage combinations, the most effective phages were combined with one of either of the two less effective phages (Fig. 4E and F).

The best combination of phages for the lysis of CD105HE1 was phiCDHM1-phiCDHM2-phiCDHM3, which eliminated all viable bacteria by 2 h postinfection. Both the phiCDHM1-phiCDHM5-phiCDHM6 and phiCDHM2-phiCDHM5-phiCDHM6 combinations produced an approximately 4-log-unit reduction by 24 h (Fig. 4E). Of note, the four-phage combination, phiCDHM1-phiCDHM2-phiCDHM5-phiCDHM 6, resulted in a 6-log-unit reduction in bacterial recovery, but this was not as efficient as the best three-phage combination.

The best three-phage combination for CD105LC2 was also phiCDHM1-phiCDHM2-phiCDHM3, which completely cleared the cultures in 5 h, whereas phiCDHM1-phiCDHM5-phiCDHM6 completely cleared the cultures in 24 h. phiCDHM2-phiCDHM5-phiCDHM6 was less effective and reduced the counts of the CD105LC2 cultures to approximately 10^2^ CFU/ml after 5 h, after which growth appeared to be stationary (Fig. 4F). However, the most effective combination for this strain was the four-phage combination, phiCDHM1-phiCDHM2-phiCDHM4-phiCDHM6, which cleared the bacterial culture after 3 h with no further regrowth.

### Sensitivity of phage-resistant and lysogenic CD105LC2.

To determine why some phage combinations were more effective than the individual phage treatments, resistant and lysogenic bacteria were recovered following treatment with phiCDHM1, phiCDHM2, or phiCDHM3. Primers specific for individual phages were used to establish that the recovered bacteria were genuinely phage resistant or lysogens. The sensitivity of these strains to each of the individual infecting phages was retested, and their sensitivities compared to those of untreated bacteria were determined. The results showed that the phage-resistant and lysogenic isolates were no longer susceptible to the phage against which they had been tested (except for the lysogenic CD105LC2 strain tested against phiCDHM2). However, they were still susceptible to at least two of the other phages available in the test pool (Table 2).

**TABLE 2:** Susceptibility of phage-resistant bacteria and lysogens to phage infection^*a*^

CD105LC2 strain	Phage titer (PFU/ml)				
	phiCDHM1	phiCDHM2	phiCDHM3	phiCDHM5	PhiCDHM6
WT	2.0 × 10^9^	1.5 × 10^9^	4.0 × 10^9^	3.0 × 10^9^	2.0 × 10^9^
RphiCDHM1	0	4.0 × 10^3^	1.0 × 10^6^	2.0 × 10^9^	2.5 × 10^9^
LphiCDHM1	3.25 × 10^4^	0	2.5 × 10^8^	4.0 × 10^9^	4.25 × 10^9^
RphiCDHM2	3.0 × 10^3^	0	1.5 × 10^7^	7.25 × 10^7^	6.5 × 10^6^
LphiCDHM2	2.5 × 10^3^	3.0 × 10^3^	2.5 × 10^9^	3.5 × 10^9^	1.5 × 10^9^
RphiCDHM3	1.5 × 10^7^	1.0 × 10^7^	0	0	0

a The susceptibility to phiCDHM1 to phiCDHM3, phiCDHM5, and phiCDHM6 infection was determined using plaque assays on phage-resistant bacteria (RphiCDHM1, RphiCDHM2, and RphiCDHM3) and lysogens (LphiCDHM1 and LphiCDHM2). The results obtained were compared with those for the wild-type (WT) bacteria.

### Effects of phage-phage interactions in combinations and when used in mixed ribotypes *in vitro*.

To determine whether phages had synergistic or antagonistic effects on each other, a mixture which combined the three most active myoviruses with the siphovirus phage phiCDHS1 was prepared. Phage phiCDHS1 shows particular specificity for the epidemic 027 ribotype (of which strain R20291 is frequently used as the exemplar). Infection with this phage resulted in a 5-log-unit reduction in the viability of R20291 at 5 h postinfection, with no subsequent change in bacterial counts being observed up to 24 h (see Fig. S2 in the supplemental material). Combining this phage (phiCDHS1) with the other myoviruses had no antagonistic effects on its activity, with the reduction of R20291 being proportional to the quantity of phiCDHS1 in the culture.

As infection with multiple ribotypes has been reported (56), we tested the ability of the phages to target a culture containing two ribotypes (CD105LC2 ribotype 014/020 and R20291 ribotype 027). The mixed culture was treated with a combination of phiCDHM1-phiCDHM2-phiCDHM3 (targeting CD105LC2) and phiCDHS1 (targeting R20291). A 7-log-unit reduction in the viability of the total C. difficile population was observed at 4 h posttreatment, although intact bacteria (10^2^ CFU/ml) were isolated by 20 h later (see Fig. S3 in the supplemental material). Using bacteria recovered from the 24-h culture, 80 colonies were subjected to ribotype analysis, which revealed that only 2 (2.5%) of the colonies were of the 014/020 ribotype. The relatively low proportion probably reflects the presence of several phages that specifically target this strain, thus reducing the opportunity for resistance to develop.

### Impact of phage combinations on C. difficile *in vivo*.

To establish the suitability of these phages for therapeutic purposes, we first determined how effective they were in a hamster model of CDI. To allow direct comparisons between the *in vitro* and *in vivo* data, we initially determined the virulence of strain CD105HE1 in the hamster model. Infection with 0.2 ml of 10^4^ CFU/ml CD105HE1 spores resulted in a typical profile of C. difficile disease, with all animals displaying classical symptoms of infection (a wet tail and a drop in the core body temperature to <35°C). All animals were culled at the established humane endpoint, approximately 55 h postinfection. This time to the endpoint was similar to that for other C. difficile strains, including CD630 Δ*ermB* (44 h; range, 40 to 48 h) and R20291 (47 h; range, 35 to 62 h) (see Table S4 in the supplemental material). On postmortem examination, all animals showed high levels of colonization and significant levels of spores and toxin B within the cecum and colon (see Fig. S4A and B in the supplemental material), where the bacteria are known to proliferate and tissue damage is most significant.

**FIG 5:**
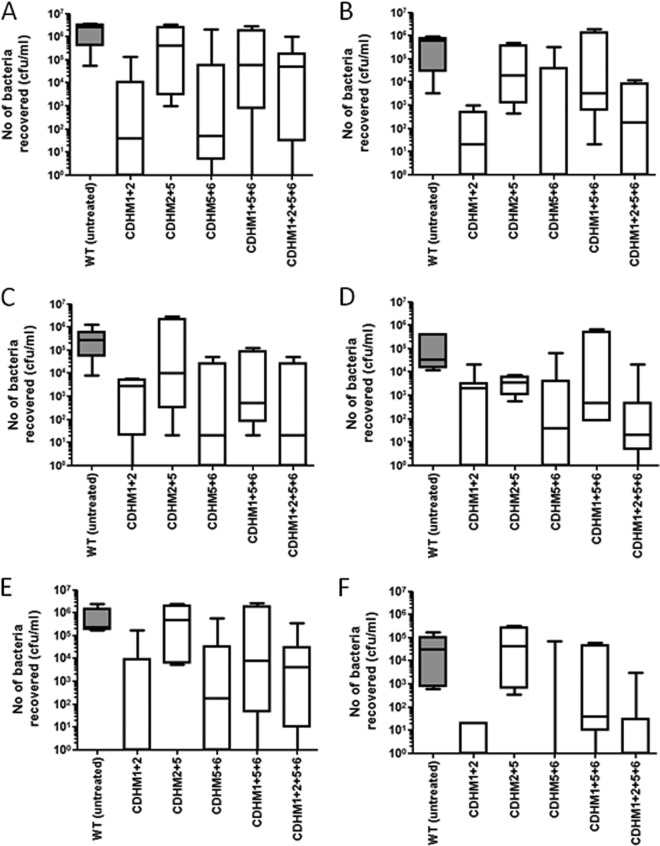
Levels of colonization in animals treated with different phage combinations and culled at 36 h postchallenge with CD105HE1. Changes in vegetative cell populations (A to D) and changes in relative levels of recovered C. difficile spores (E and F) are shown. (A to D) The average total number of LA bacteria recovered from the cecal contents (A), LA bacteria recovered from the colon (B), TA bacteria recovered from the cecum (C), and bacteria associated with the colon (D) are presented. (E and F) The number of heat-resistant (spores) LA bacteria from the cecum (E) and the number of spores recovered from the lumen of the colon (F) are also shown. The median level of colonization is represented, while boxes indicate the upper and lower quartile spread of colonization within the group. The whisker bars represent the highest and lowest levels of colonization observed from data generated from a minimum number of four animals for each treatment. WT, wild type.

To determine how effective the phages were at reducing colonization, the animals were treated with 10^4^ CFU/ml spores and 10^8^ PFU/ml individual phages or phage combinations. At every 8 h postinfection, animals were additionally given 10^8^ PFU/ml individual phages or phage combinations, with the animals being culled at 36 h postinfection (Fig. 1A). This time point was chosen because in untreated animals, toxin production in the cecum and colon is limited (with little or no change to Vero cells being observed when they are treated with dilutions of filtered gut samples), while the recovery of bacteria is consistently high. This allowed us to determine the effects of the phages on colonization in the absence of toxin-related symptoms. Using the *in vitro* data and preliminary experimental data from studies with hamsters infected with individual phages, five different combinations of phages were chosen and tested *in vivo*. At 36 h, untreated animals showed consistently high levels of bacteria and colonization in the lumen of the cecum and colon (between 10^6^ and 10^7^ CFU/ml), with 10^4^ to 10^5^ CFU/ml being more intimately associated with the intestinal epithelial barrier. In animals treated with phages, the recovered C. difficile CFU counts were significantly lower in these tissues (Fig. 5A to F). These data show that the most effective treatment combinations were phiCDHM1-phiCDHM2 and phiCDHM5-phiCDHM6. These treatments reduced the bacterial counts from the lumen samples by at least 4 log units and those from the tissue samples by approximately 2 log units. In addition, treatment with the four-phage combination (phiCDHM1-phiCDHM2-phiCDHM5-phiCDHM6) showed levels of reduction of bacterial counts similar to those achieved with the two-phage combinations. For at least one animal in each treatment group, C. difficile was undetectable in various parts of the tissue, while in others, 10^4^ or 10^5^ CFU/ml per tissue was recovered (Fig. 5A to F).

To determine whether these differences were due to the development of phage resistance, 20 individual isolates from the cecum and colon of treated animals were characterized using multilocus variable-number tandem-repeat analysis typing (57) (see Fig. S5 in the supplemental material). These molecular profiles confirmed that the recovered bacteria were CH105HE1, and in each case, these bacteria remained sensitive to the phages used in the treatment. This suggests that the infection outcome may be influenced by the ratio of phage/bacteria during the initial phase of bacterial outgrowth.

### Impact of phage combinations on C. difficile sporulation and toxin production *in vivo*.

To determine whether treatment influenced the levels of detectable toxin produced by the bacteria *in vivo*, gut filtrates were evaluated for toxin B activity on Vero cells and toxin A activity on HT-29 cells. As predicted, there was very little evidence of the presence of active toxin in any of the gut washes (no cell rounding was observed when filtered gut contents diluted 1:10 were added) at this time point even in untreated animals. This supports previous observations that toxin is detected only approximately 6 h prior to a drop in body temperature, which would be significantly later (approximately 49 h) than the 36-h time point used here (58).

The amount of spores recovered from animals treated with these phage combinations was also significantly lower than the amount recovered from control animals. In the cecum, these numbers dropped by approximately 1 log unit in treated animals compared to the numbers in untreated animals. The drop in the colon was greater, dropping from 4 log units in untreated animals to 2 log units in treated animals (*P* = 0.011 and *P* = 0.004 for cecal and colon lumen-associated bacterial spores, respectively).

### Impact of phage combinations on C. difficile *in vivo* in an endpoint model.

**FIG 6:**
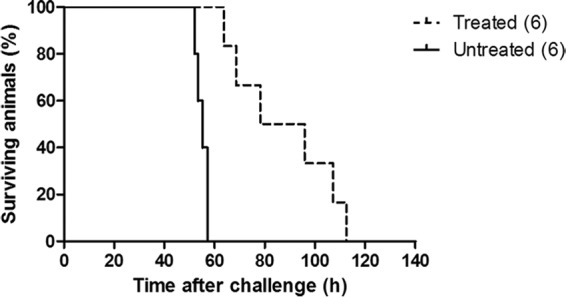
Impact of treatment on survival of animals challenged with CD105HE1. Untreated animals reached the experimental endpoint earlier than animals treated with bacteriophage preparation phiCDHM1-phiCDHM2-phiCDHM5-phiCDHM6. Treated animals received 14 doses of bacteriophage preparations every 8 h from the point of challenge to the clinical endpoint. A statistically significant difference in the time to the experimental endpoint (*P* = 0.0007) was determined using group sizes of six animals each.

To further evaluate the efficacy of phage therapy on disease severity, hamsters were infected as described above and treated with phage every 8 h until the endpoint of the experiment (Fig. 1B). The animals were monitored for symptomatic disease and culled at the humane endpoint described above. Untreated animals infected with CD105HE1 reached this experimental endpoint at approximately 55 h postinfection, but infected animals treated with the four-phage combination (phiCDHM1-phiCDHM2-phiCDHM5-phiCDHM6) had a clear delay to the experimental endpoint (87 h 40 min ± 20 h 28 min) (Fig. 6). This delay was statistically significant (*P* = 0.0007) between the treated and untreated groups, despite the variation in the time to the onset of symptoms within the treated group.

## DISCUSSION

The need for alternative treatments for C. difficile infection has been widely discussed. In a recent review, Zucca et al. analyzed all the existing and new developments in this field and highlighted the key advantages which a bacteriophage-based approach would offer (12). These include avoidance of microbiome dysbiosis, bacterium/host specificity, tolerance by patients, and a lack of interference with antibiotics (12, 14, 59). Here, we present data on the characterization of seven phages of C. difficile and describe their ability to lyse the bacteria and potentially treat CDI in hamsters. In particular, combinations of phages optimized *in vitro* were shown to be effective in the treatment of C. difficile infection *in vivo*.

While phages with lytic activity against C. difficile have been described previously (14, 29), their direct therapeutic application has been limited by the poor availability of multiple phages that target this pathogen. No strictly virulent C. difficile phages have been isolated, despite the efforts of several research groups (60–62). This has hampered their development for therapeutic use, as temperate phages are considered undesirable due to their capacity to transduce or lysogenize infected bacteria (30, 31, 34). In reality, many phage mixtures effective against other target bacteria have been produced, and in those studies, phages were selected solely on the basis of their activity *in vitro* and *in vivo* (25) and knowledge of the encoded integrase genes was limited. The phages described in this study were not strictly virulent, as despite their lytic activity, their genomes encode integrases. The absence of C. difficile phages that lack such integrases and the clear lytic properties of the phages in our collection on a broad range of clinical strains suggest that the information obtained for these phages provides a strong foundation of knowledge from which further improvements can be made.

When they were combined, the phages described had lytic activity on 86% of the clinically relevant ribotypes (18 of the 21 ribotypes tested). We showed that the phages could lyse 12 of 13 of the ribotypes that are the most prevalent in the United Kingdom (63), thus having 92% coverage of such strains, and had 100% killing efficacy against the emerging ribotypes (ribotypes 002, 005, 014/020, 015, and 078) that are increasingly causing concern in the United Kingdom and the United States (6, 64, 65). These ribotypes have also been shown to be associated with increased disease severity and fluoroquinolone resistance (51, 66).

As multiple phages are needed to target the wide range of strains capable of causing clinical disease, the phages were tested in combination with each other. Phage cocktails have previously been shown to be effective in the treatment of Pseudomonas aeruginosa, Klebsiella pneumoniae, Vibrio cholerae, Clostridium perfringens, and E. coli in animal models. In all the cases mentioned above, phage cocktails have been shown to either reduce the bacterial load, resulting in full recovery; cause a delay to the endpoint; or reduce the rate of mortality among the animals (25). The approach of using phage cocktails is also known to limit the buildup of resistance and could curtail any problems with phage lysogeny (25). In order to select these combinations, phages that had diverse morphologies and infectivity profiles were chosen.

Fundamentally, the efficacy and usefulness of a particular phage depend upon its host range, with those able to lyse a wide range of ribotypes being the most suitable for therapeutic purposes. In this study, six of the phages tested individually showed lytic activity on C. difficile isolates belonging to more than 7 different ribotypes, with phiCDHM3 showing lytic activity on isolates of 12 ribotypes. In contrast, phiCDHM4 lysed isolates from 4 ribotypes but was the only phage that showed any activity against isolates of ribotype 012. Similarly, inclusion of phiCDHS1 should be considered in any future combination established for clinical use, as this was the only phage to infect ribotype 027 and 001 isolates (Fig. 3A and B). These data support the idea that an effective therapeutic approach will require a combination of phages and that inclusion of some narrower-host-range phages is necessary to ensure good coverage (41).

In addition to host range, phages need to have robust lytic properties, and extensive characterization of these activities using individual phages provided the basis on which the most effective phage combinations were identified. The capacity of phages to completely clear cultures of C. difficile was variable, as previously reported (37, 61, 67), and regrowth of bacteria was observed in all cases where phages were administered individually. At present, it is unclear whether this reflects immunity as a consequence of lysogeny to superinfection by the same phage (30, 68) or the development of phage resistance (14). However, the fact that recovered bacteria were still sensitive to other phages in the cocktail supports the premise that multiple-phage combinations are required for effective clearance. Regrowth could additionally reflect the changing physiological conditions of the bacteria within the culture, which may impact the phage absorption rate, the ability of the phages to access the bacterial genome, and the burst size (69). Interestingly, bacteria recovered *in vivo* were still sensitive to infection by the phages found in the treatment combinations. This suggests that the surviving bacteria *in vivo* had not encountered the phages either when the phages were present in numbers sufficient to ensure complete lysis or at a time when the bacteria were physiological insensitive to infection (70, 71).

When the phages were combined, C. difficile clearance was more effective and the extent of regrowth was either completely eliminated or significantly reduced. This is consistent with observations in other bacterial systems (25) and with our data showing that the phage-resistant bacteria were still susceptible to other phages tested (Table 2). This observation has been reported for Lactococcus lactis phage c2, which has been shown to infect lysogens that were created by exposure to a different phage (72).

From these data, it would appear that the effectiveness of different phage combinations is likely to be influenced by the sensitivity of the infecting strain. This is illustrated by the fact that the three-phage combination was more effective at clearing CD105HE1 (ribotype 076), while a four-phage combination was more efficacious against CD105LC2 (ribotype 014/020). This suggests that the phage formulation may need to be adjusted to reflect the targeting of dominant ribotypes (12, 14).

Within the hamster model of infection, we observed that high titers (10^7^ PFU/ml) of infectious phages could be recovered from the relevant sites of C. difficile colonization, the cecum and colon. The number of bacteria recovered from animals treated with combinations of phages was reduced at 36 h (Fig. 5). Treatment with the three-phage combination phiCDHM2-phiCDHM5-phiCDHM6 *in vivo* was less effective than treatment with any of the two-phage combinations tested. These data suggest that, at least for CD105HE1, the phage phiCDHM1 plays an important role in clearance, as combinations that included this phage were the most effective *in vivo*. Importantly, as the number of phages in the combination increased, the MOI of the individual phages in the preparation was reduced. Thus, the three-phage combination may have been less effective, as addition of a third poorly effective phage may have reduced the concentration of the effective phage to a suboptimal concentration within these experiments. In contrast, inclusion of four phages was highly effective and probably reflects the lytic activity of phiCDHM1, even when it was given at a lower dose. Further optimization of the MOIs of individual phages within this combination should generate a more effective and optimal treatment. A major highlight of this work is the correlation between the observations made *in vitro* and the corresponding levels of colonization in the animal models. This gives confidence that the phage combinations tested against other strains, including CD105LC2 and R20291, will also be effective *in vivo*.

In the final animal experiment, using the endpoint regime, we observed a delay of approximately 33 h between the average time to the endpoint calculated for the untreated and treated animals. Although complete protection was not observed, this delay to symptom onset is consistent with other published data (25). While single phages have previously been used to treat CDI in animal models, in both these published studies, vegetative cells were used to establish infection (29, 30). In those studies, animals were treated with phage within 1 h of infection with vegetative cells (29, 30). Using this treatment regimen, it is possible that the phage could infect and destroy the majority of bacteria during transit to the cecum and colon. In contrast, animals in this study were infected with a combination of spores and phages. Evidence suggests a role for conjugated primary bile acids in C. difficile germination (73), and therefore, it is unlikely that C. difficile is sensitive to phage until the spores are exposed to bile in the small bowel. Using this approach, the interaction between phages and susceptible germinating vegetative cells is likely to be limited, with variable numbers of infected and uninfected vegetative cells entering the cecum and colon (25, 71). This could lead to the different levels of colonization and disease observed. Interestingly, lower numbers of spores were recovered from the cecum and colon of animals receiving the most effective treatments (phiCDHM1-phiCDHM2, phiCDHM5-phiCDHM6, and phiCDHM1-phiCDHM2-phiCDHM5-phiCDHM6). As sporulation is generally a feature of the final stages of infection in animals, these results suggest that infection had not progressed as rapidly within these animals. This observation was further supported by the fact that treated animals showed a significantly longer time to reach the clinical endpoint.

Further work currently being undertaken will determine whether phage combinations are effective for the treatment of low-level colonization. Clearly, it is important to explore how phages can be modified to improve their activity. This includes the removal of identified integrases to prevent future integration and increase burst size. In addition, it will be useful to establish whether phages can be used as an adjunct to antibiotic therapy, with their administration alongside or after antibiotic treatment further reducing the potential of infection relapse. While the *in vivo* analysis was performed with only one strain, the correlation between lysis activities *in vitro* and *in vivo* gives confidence that this approach can be applied more generally. Together these data provide evidence that phage treatment is a valid approach toward limiting C. difficile colonization and suggest that this may be a useful tool in the fight against this infection in the future.

## Supplementary Material

Supplemental material
