# Supplementary material for: Bacteriophage Combinations Significantly Reduce Clostridium difficile Growth In Vitro and Proliferation In Vivo
Source: Antimicrob Agents Chemother. 2016 Jan 29;60(2):968–81. doi: 10.1128/AAC.01774-15 (PMC4750681; doi:10.1128/AAC.01774-15)
Supplement: Supplemental material [file supp_60_2_968__index.html]

Bacteriophage Combinations Significantly Reduce Clostridium difficile Growth In Vitro and Proliferation In Vivo — Supplemental material 

# Bacteriophage Combinations Significantly Reduce Clostridium difficile Growth *In Vitro* and Proliferation *In Vivo*

## Supplemental material

- Supplemental file 1 -

  Supplemental Figures S1 to S5 and Tables S1 to S4.

  PDF, 394K
