# Supplementary material for: Bacteriophage Combinations Significantly Reduce Clostridium difficile Growth In Vitro and Proliferation In Vivo
Source: Antimicrob Agents Chemother. 2016 Jan 29;60(2):968–81. doi: 10.1128/AAC.01774-15 (PMC4750681; doi:10.1128/AAC.01774-15)
Supplement: Supplemental material [file AAC.01774-15_zac002164838so1.pdf]

SUPPLEMENTARY MATERIALS

Table S 1 Accession numbers of all phage sequences examined in this study

Name	Phage morphology	Accession numbers
**phiCDHM14**	Small myovirus	LK985321
**phiCDHM11**	Small myovirus	HG798901
**phiCDHM13**	Small myovirus	HG796225
**phiMMP04**	Small myovirus	NC_019422.1
**phiCDHM1***	Medium myovirus	HG531805
**phiCDHM3***	Medium myovirus	LN680004
**phiC2**	Medium myovirus	NC_009231.1
**phiCD119**	Medium myovirus	NC_007917.1
**phiCDHM19**	Medium myovirus	LK985322
**phiCDHM2***	Long tailed myovirus	LN680003
**phiCDHM4***	Long tailed myovirus	LN680005
**phiCDHM5***	Long tailed myovirus	LN680006
**phiCDHM6***	Long tailed myovirus	LN680007
**phiCD27**	Long tailed myovirus	NC_011398.1
**phiMMP02**	Long tailed myovirus	NC_019421.1
**phiCDHS1***	Siphovirus	LN680008
**phiCD38-2**	Siphovirus	NC_015568.1
**phiCD6356**	Siphovirus	NC_015262.1

*****phages tested in this study.

Table S 2 Analysis of manufacturing hosts for the expression of lysogenic phages

	Manufacturing host	Morphology of prophage	Prophage inducing agent	TEM image of prophage
**1**	CD105HE1	Myovirus	Mitomycin C/Norfloxacin	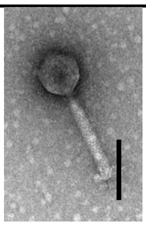
**2**	CD105LC1	Defective myovirus	Mitomycin C	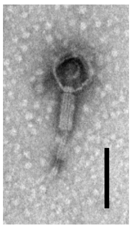
**3**	CD105HS1	Phage tail-like particle	Mitomycin C/Norfloxacin	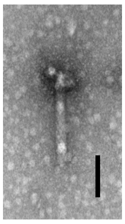

Mid-log phase cultures of bacteria were treated with prophage inducing agents, mitomycin C or norfloxacin (3 µl/ml) to mediate prophage release. Phages present were analyzed using transmission electron microscopy. Bar ~100 nm.

.

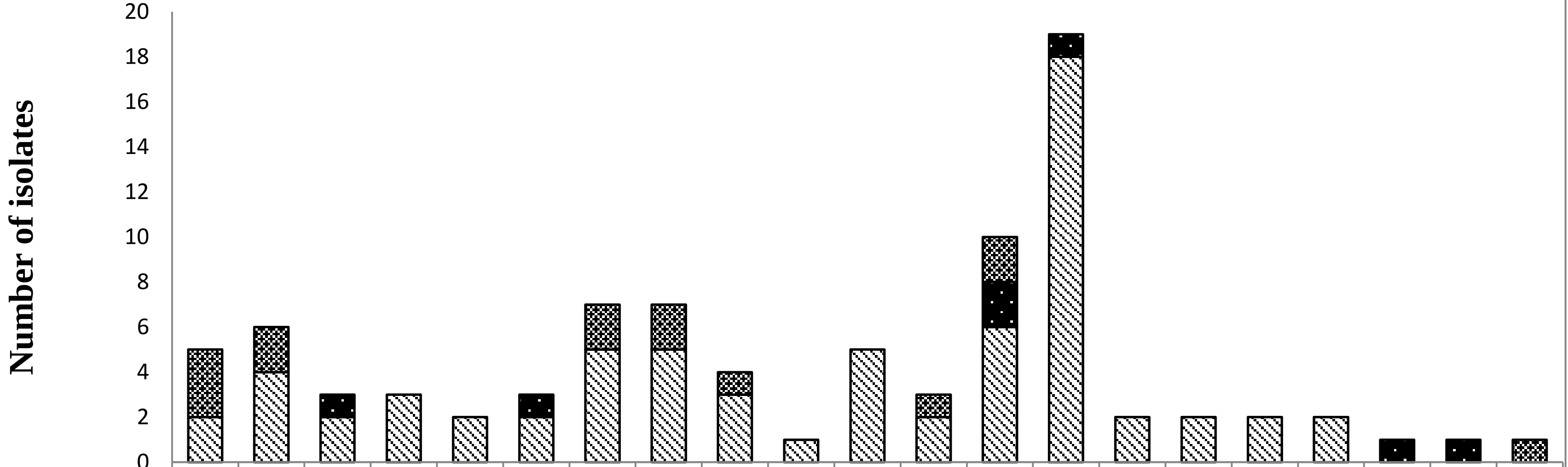

	R001	R002	R003	R005	R012	R013	R014/ 020	R015	R017	R018	R023	R026	R027	R078	R081	R087	R106	R107	R126	R262	R127
AUSTRALIA	3	2	0	0	0	0	2	2	1	0	0	1	2	0	0	0	0	0	0	0	1
USA	0	0	1	0	0	1	0	0	0	0	0	0	2	1	0	0	0	0	1	1	0
UK	2	4	2	3	2	2	5	5	3	1	5	2	6	18	2	2	2	2	0	0	0

Figure S 1 Number of ribotype isolates obtained from the different countries.

Distribution of the 80 *C. difficile* isolates representing 21 clinically relevant ribotypes obtained from the UK, USA and Australia. The proportions of isolates from each ribotype and country are represented.

Table S 3 Host range analysis of the phages examined in this study

Ribotypes	Bacterial isolates	Phages						
		phiCDHM1	phiCDHM2	phiCDHM3	phiCDHM4	phiCDHM5	phiCDHM6	phiCDHS1
001	AUS1025							
	AUS1021							
	AUSCD84							
	AIP							
	CD001							
002	AUS1033							
	AUS1036							
	AIL							
	AIJ							
	TL178							
	ATH							
003	LEEDS003							
	AQV							
	2007831							
005	AUE							
	AOY							
	AOO							
012	CD630							
	10							
013	ASA							
	AKR							
	ARS							
014/020	ATJ							
	CD105LC2							
	ATK							
	TL176							
	AUS1022							
	CD66							
	ANS							
015	TL174							
	ALV							
	ATO							
	ATU							
	ATR							
	CD89							
	CD81							
017	AUS1023							
	CF5							
	M68							
	LEEDS017							
018	LEEDS018							
023	CD305							
	UK023							
	AKL							
	AJX							
	LEEDS023							
026	AUS1028							
	ALN							
	AUB							

Table S 3 continue

Ribotypes	Bacterial isolates	Phages						
		**phiCDHM1**	**phiCDHM2**	**phiCDHM3**	**phiCDHM4**	**phiCDHM5**	**phiCDHM6**	**phiCDHS1**
027	AUS1032							
	AUS1024							
	CD196							
	R20291							
	B19							
	AJS							
	AJV							
	AMJ							
	AMZ							
	ANB							
	US027							
	2006237							
078	M120							
	ATM							
	AKM							
	AKP							
	ALL							
	ARS							
	AML							
	5361							
081	ANO							
	ANQ							
087	APX							
	APT							
106	CD106							
	LV22							
107	ASV							
	ASQ							
126	2005093							
127	M322630							
262	2007827							
**Total number of ribotypes = 21**		**7**	**9**	**12**	**4**	**10**	**9**	**11**
**Total number of bacterial isolates = 80**		**16**	**22**	**31**	**4**	**20**	**23**	**30**

Host range analysis was conducted by applying 10 µl of 10^8 PFU/ml of phage stocks on lawns of bacteria and incubated anaerobically at 37 ^{o}C for 18-24 h. Key: Red cells= clears, Purple cells =turbid clearing, white cells = no clearing, Grey cells=UK strains, Blue cells = Australian strains and Green cells= US strains.

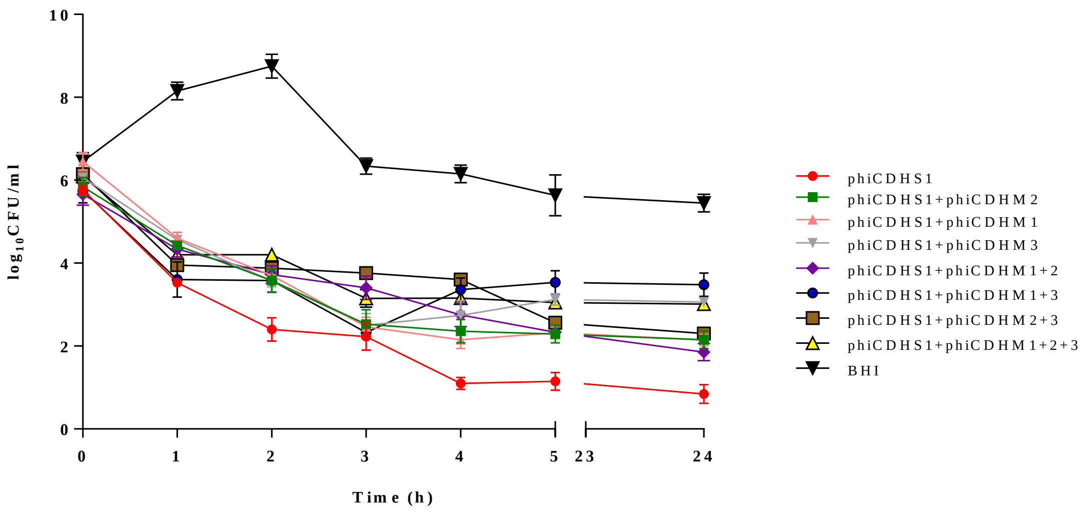

Figure S 2. Number of viable *C. difficile* R20291 following treatment with phage combinations.

Phage treatments of *C. difficile* R20291 cultures with various phage combinations are shown. Enumeration of viable bacteria in the treated and untreated cultures at the different time points was determined on BHI agar plates. Error bars represent standard error of mean (SEM) of three replicates.

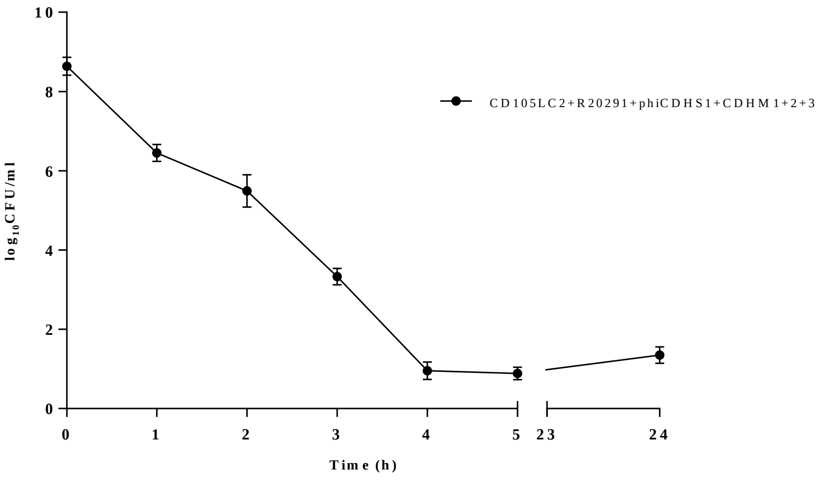

Figure S 3 Infection of R20291 and CD105LC2 cultures with phiCDHS1+phiCDHM1+2+3.

Total viable bacterial counts in the phage-treated culture medium at different time points were determined on BHI agar. Error bars represent standard error of mean (SEM) of three replicates.

Table S 4 Times to temperature drop of 35°C and humane endpoint cull for hamsters infected with different strains of *C. difficile*.

Strain tested	Ribotype	No of animals tested	Average time to 35°C temp drop	Range
**R20291**	RT027	11	46 h 42 m	36 h - 62h 18 m
**CD630 ΔermB**	RT012	6	53h 39 m	34 h 54 m – 67 h 36 m
**CD105HE1**	RT076	6	55 h 31 m	52 h 3 min - 58 h 20 m

These infections were initiated 5 days post treatment with clindamycin as all strains show sensitivity to clindamycin used to modify gut flora.

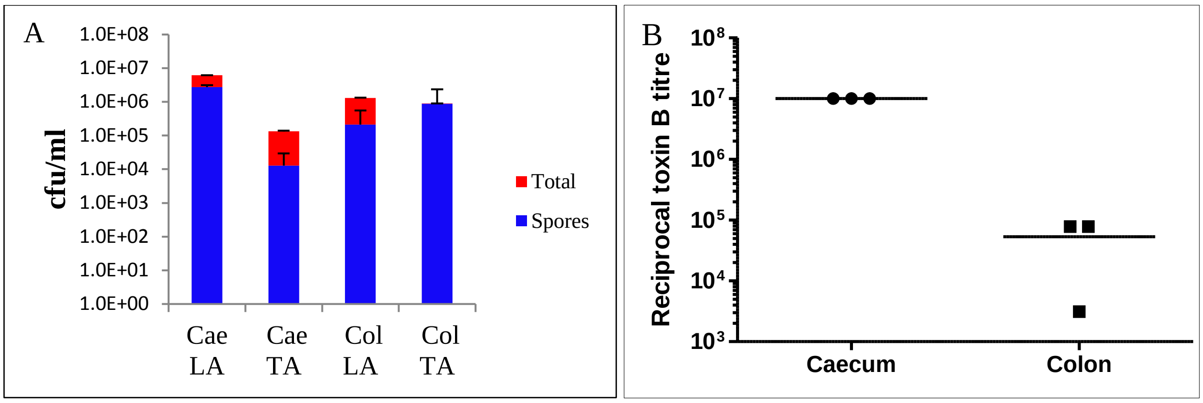

Figure S 4 Levels of CD105HE1 colonization and toxins at experimental endpoint in hamsters treated with the bacteria alone

A, Recoveries of CD105HE1 at experimental endpoint from hamsters. The caecum (Cae) and colon (Col) of each animal was opened longitudinally and washed thoroughly to remove the luminal associated organisms (LA). The tissue was then homogenized to allow quantification of tissue associated (TA) organisms. Levels of colonization were determined before and post heat treatment (56°C for 20 min) to allow discrimination of total (red bars) or spore counts (blue bars) respectively. Error bars represent the standard error of mean bacterial recoveries from six animals. **B, Assessment of Toxin B levels in filtered gut extracts from hamsters at experimental endpoint**. Diluted filtered contents from the caecum and colon of infected animals were applied to confluent wells of Vero cells and activity of the toxin determined 24h later. The reciprocal of the first dilution that showed < 50% cell rounding was recorded as activity. Values shown reflect a single experimental evaluation of 3 of the 6 animals tested.

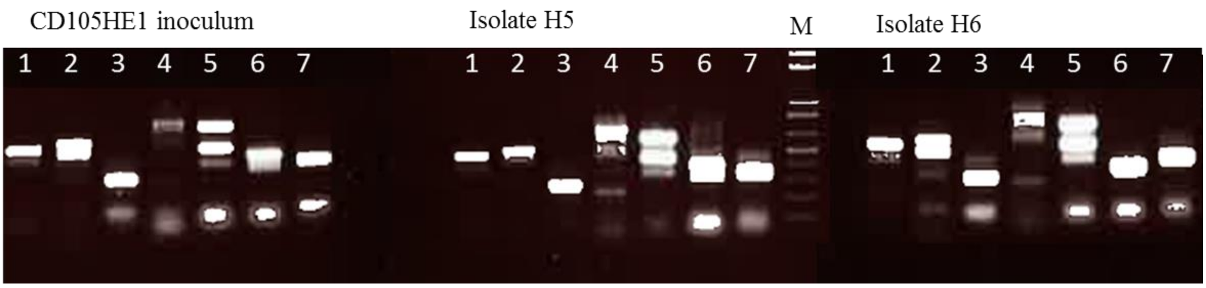

Figure S 5 Confirmation that *C. difficile* recovered from phage treated hamsters was CD105HE1.

Analysis is based on the use of seven primers directed toward multilocus variable-number tandem-repeats sequences (MLVA)[70] to amplify specific sequences from DNA recovered from organisms used to challenge animals and for colonies recovered from the caecum and colons of treated animals. Two representative isolates from two H5 and H6, challenged with CD105HE1 and treated with phage cocktail are shown here. M is 1 kb Plus DNA ladder (Invitrogen, USA).
